# Epidemiological behavior of suicide attempt in Colombian adolescents years 2016-2019: An ecological study

**DOI:** 10.1590/1518-8345.6240.3807

**Published:** 2022-11-25

**Authors:** Lauren Camila Murillo Gutiérrez, Mónica Paola Quemba Mesa, Leidy Yemile Vargas Rodríguez, Isabel Cristina Florez Escobar, José Ivo Contreras Briceño

**Affiliations:** 1 Universidad de Boyacá, Programa de Enfermería, Tunja, Boyacá, Colombia; 2 Universidad Técnica Particular de Loja, Carrera de Enfermería, San Cayetano Alto, Loja, Ecuador

**Keywords:** Attempted Suicide, Adolescent, Morbidity, Epidemiology, Nursing, Colombia

## Abstract

**Objective:**

characterize the behavior of attempted suicide in adolescents in Colombia, and its associated epidemiological factors from 2016 to 2019.

**Method:**

quantitative, observational, descriptive, and ecological study. The sample was extracted from the database of the Integrated Social Protection Information System, by identifying cases of attempted suicide for ages in the range from 12 to 17 years old, calculated for a prevalence estimate based on a total population of 32,076. Univariate and bivariate analysis of the variables of interest was performed.

**Results:**

suicide attempts occurred more frequently in females (n: 24,619; 76.7%), of subsidized regime (n: 17,960; 56%); and being in psychiatric centers is the most frequent vulnerability condition (n: 676; 2.11%). Living in the capital city is a risk factor for attempted suicide (OR: 1.423; 95% CI: 1.385 to 1.462), while being male is a protective factor (OR: 0.290; 95% CI: 0.283 a 0.298).

**Conclusion:**

suicide attempts are a subject of interest in Public Health due to their prevalence and impact on the family and social environment. The Nursing professional is competent in the identification, treatment, and prevention of this phenomenon.

## Introduction

Suicide is a phenomenon that begins with suicidal ideation and intention until its consummation. In this context, suicide attempt presents a set of behaviors initiated by the subject who, in carrying them out, has at least some intention to die, although these may or may not cause medical injuries^1^. According to the World Health Organization (WHO), there are many suicide attempts for every completed suicide and, in the population at large, an unfinished suicide attempt is the single most important risk factor^2^.

In this perspective, suicide is the fourth leading cause of death among young people aged 15-19 years old, and rates are higher among vulnerable and discriminated groups such as refugees and migrants, indigenous peoples, lesbian, gay, bisexual, transgender and intersex persons^2^. According to data from the Colombian National Institute of Health (INS) until the beginning of September 2021, there has been an increase in the rate of attempted suicide compared to the immediately preceding year^3^.

Similarly, it is possible to identify risk factors associated with suicidal ideation in adolescents in Colombia, which are related to aspects such as history of bullying, physical or sexual abuse, mental or physical illness, academic losses, unstable love relationships, difficulty tolerating economic problems, and social accommodation, abuse of psychoactive substances and intrafamily dysfunction^4^.

In this sense, it is evident that the problem has not yet been addressed in the historical-retrospective or academic context with a nursing mission approach. Even when there are research advances at the psychosocial level, these are only used as a reference for the Nursing professional, addressing social, medical, biological and scientific aspects contemplating a holistic approach to the person prone to suicide.

However, the nursing professional plays a very important role in providing humanized and holistic care with a scientific basis^5^, in addition to bearing effective competencies for prevention applied through health education. Therefore, approaching adolescents to inform and reduce the risk factors for suicide attempts would reduce this increasing social problem. Likewise, within the group of healthcare professionals, Nursing maintains direct contact with the patient, since the therapeutic relationships established are usually very close, making easier to learn about the concerns and emotional condition of adolescents^6^.

Likewise, there is significant interest and concern by healthcare professionals, particularly Nursing professionals in addressing, preventing and intervening in suicidal behavior. This is evidence of an absolute consideration of the need for care^7^. In this light, Nursing professionals trained in the area of mental health have the possibility of performing timely detection of risk factors for suicidal ideation and behavior in adolescents, in addition to proposing relevant and immediate interventions that prevent a fatal trigger^8^.

Nursing is thus recognized as a fundamental pillar in interventions at the institutional and community level, evidencing the growing need for education and training in mental health, especially in suicidal ideation and behavior. For that, there is a need for the implementation of protocols, courses of action and research aimed at healthcare personnel, teachers and parents who remain closer to adolescents and have greater possibilities for early detection of suicidal behavior in this population^8^.

Therefore, the ecological study of the epidemiological behavior of suicide attempts becomes a contribution to suicide prevention since, as the nursing professional applies the knowledge derived from research evidence in practice, it enables quality intra- and interdisciplinary intervention in the immediate care and follow-up of adolescents, as it provides a broader perspective on the prevalence and some factors related to the topic^8^. For this reason, the aim of this study is to characterize the behavior of attempted suicide in adolescents in Colombia and the related epidemiological factors, from 2016 to 2019.

## Method

### Type of study

A quantitative, observational, descriptive and ecological study was performed.

### Population, sample and sampling

Of census type, taking the totality of suicide attempt cases reported registered in the platform of the Integrated Social Protection Information System (SISPRO) of Colombia from 2016 to 2019, for a total of 32,076 cases; intentional sampling at convenience.

### Selection criteria

Cases of attempted suicide reported and registered by SISPRO, involving male and female adolescents, aged from 12 to 17 years, in 2016 and 2019 in Colombia were included. Reports with incomplete information in the databases were excluded.

### Variables of interest

From the SISPRO server in remote connection with previously acquired credentials, the following variables of interest for the event of suicide attempt were filtered, downloaded and organized in a Microsoft Excel 2016 database: age in completed years, year of report, biological sex, health regime, origin, ethnicity, geographic area and presence of conditions of vulnerability (e.g. displaced persons, victims of armed violence, migrants, etc.). The access to this server is open, as these are data of public access at the national level, and only require free prior registration to obtain login credentials.

### Analysis planning

The analysis consisted of two phases, one univariate and the other bivariate. The univariate phase allowed each variable of interest according to its nature to be reviewed by means of frequency measures (percentages with their relative and absolute frequencies) and central tendency - dispersion (for quantitative variables). Moreover, tables and schemes were developed to present data in a more organized way. In addition, population prevalence was calculated using the population projections of the National Administrative Department of Statistics (DANE) as reference.

The bivariate phase allowed the calculation of crude risk estimators of the Odds Ratio (OR) type with significance when obtaining a p less than 0.05, and with their confidence intervals at 95%. In the development of this phase, general population data were compared with the data obtained from the population with suicide attempt in these same age groups. This statistical processing was performed using the free statistical program OpenEpi.

Finally, compliance with the requirements for the development of multivariate analysis of logistic regression model type (global adjustment, goodness of fit and coefficients of determination) was verified, given that this analysis allows us to evaluate the multicollinearity of variables of interest, and control confounding bias.

### Bias control

The applicable criteria for an observational ecological study from the Strengthening the Reporting of Observational Studies in Epidemiology (STROBE) checklist were followed for the reporting of this study. To mitigate selection bias, we considered only reports with a confirmed suicide attempt reported in the SISPRO server, and cases that before being made public undergo a process of validation of the information at the level of the Colombian Health System.

Regarding the information and confounding bias related to the scarce information available and the difficulty of controlling possible confounding factors, which are part of the biases to which ecological studies are also susceptible, it is partially controlled by debugging and filtering in a unique way the complete data that did not present errors from the SISPRO server. However, limitations are recognized in the evaluation only of the reported cases and the lack of knowledge of other representative variables of the suicide attempt (family and school features, history, among others).

### Ethical considerations

Likewise, the study complies with the recommendations established in international and national regulations regarding health research; specifically, according to Resolution 8430 of 1993, articles 10 and 11 on the type and level of risk, given its nature and methodology this study is risk-free research due to the retrospective documentary research. Finally, with respect to this resolution, and according to the provisions of article 21, the authors trust in the veracity of the data and the results obtained through the SISPRO database that, for belonging to the National Government, is supposed to contain true and reliable information. The research is carried out with full respect to copyrights and the findings made in other research works, considering these as complements that support the conclusions and results of this research.

## Results

In the debugging of the SISPRO database, out of a total of 32,226 records 150 (0.4%) were discarded as they did not meet the selection criteria, did not have complete information, and had errors in the report. Univariate and bivariate analysis was performed. The statistical requirements for the development of a logistic regression model (global adjustment, goodness of fit and determination coefficients) were not met, which is considered to be a limitation of the study.

**Figure 1:**
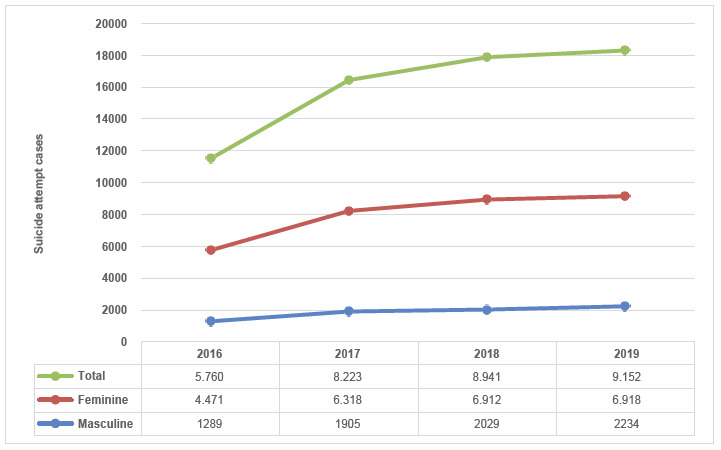
Trend of suicide attempt cases by sex in adolescents in Colombia, 2016-2019

A total of 32,076 reports of attempted suicide of adolescents aged 12 to 17 years in Colombia from 2016 to 2019 were analyzed. Reports disclose a constant increasing trend, and occur more frequently in female adolescents (n: 24,619; 76.7%) (Figure 1). This event of Public Health concern occurred more frequently in adolescents aged 15 to 17 years (n: 6,797 - 21%; n: 6.694 - 21% and n: 6,565 - 20%, respectively), and the most frequent type of scheme is subsidized (n: 17,960; 56%) (Table 1).

**Table 1:** Sociodemographic and epidemiological factors by sex in relation to suicide attempt in adolescents. Colombia, 2016-2019

Characteristic		Female n (%)	Male n (%)	Total n (%)
Year	2016	4.471(78)	1.289(22)	5.760(18)
	2017	6.318(77)	1.905(23)	8.223(26)
	2018	6.912(77)	2.029(23)	8.941(28)
	2019	6.918(76)	2.234(24)	9.152(29)
Age	12 years	1.771(81)	405(19)	2.176(7)
	13 years	3.486(86)	576(14)	4.062(13)
	14 years	4.828(84)	954(16)	5.782(18)
	15 years	5.350(79)	1.447(21)	6.797(21)
	16 years	4.813(72)	1.881(28)	6.694(21)
	17 years	4.371(67)	2.194(33)	6.565(20)
Type of regimen	Subsidized	13.830(77)	4.130(23)	17.960(56)
	Contributive	9.189(76)	2.853(24)	12.042(38)
	Special	388(79)	102(21)	490(2)
	Exception	348(78)	100(22)	448(1)
	Not associated/Undefined	864(76)	272(24)	1,136(4)
Ethnicity	Indigenous	505(68)	237(32)	742(2)
	Gypsy	69(59)	48(41)	117(0.36)
	*Raizal* (San Andrés y Providencia)	35(81)	8(19)	43(0.13)
	Palanquero from San Basilio	7(88)	1(13)	8(0.02)
	Black, mulatto, African-Colombian	988(81)	230(19)	1.218(4)
	Other ethnics	23.015(77)	6.933(23)	29.948(93)
Vulnerability conditions	Armed violence victim	110(83)	23(17)	133(0.41)
	In care of the ICBF	263(66)	134(34)	397(1.24)
	Migrants	99(76)	32(24)	131(0.41)
	Indigent Population	15(58)	11(42)	26(0.08)
	Disabled	31(61)	20(39)	51(0.16)
	Displaced	218(76)	68(24)	286(0.89)
	Demobilized	5(50)	5(50)	10(0.03)
	Incarcerated	6(19)	26(81)	32(0.10)
	Pregnant women	268(100)	0 (0%)	268(0.84)
	In psychiatric centers	516(76)	160(24)	676(2.11)
Area	Capital	19.619(77)	5.836(23)	25.455(79)
	Populated center	1.965(78)	559(22)	2.524(8)
	Remote rural area	3.035(74)	1.062(26)	4.097(13)

In relation to ethnicity, the most frequent is black, mulatto and Afro-Colombian (n: 1,218; 4%); the most frequent vulnerability conditions are being in psychiatric centers (n: 676; 2.11%) and being in the care of the Colombian Family Welfare Institute (ICBF) (n: 397; 1.24%), meanwhile, the most frequent area of residence is the municipal capital (n: 25,455; 79%) (Table 1). Living in the municipal capital is associated as a risk factor for the development of suicide attempts in adolescents (OR: 1.423; 95% CI: 1.385 to 1.462; p=0.000) and being male as a protective factor for the development of attempted suicide in adolescents (OR: 0.290; 95% CI: 0.283 to 0.298; p=0.000) (Table 2).

**Table 2:** Variables associated to suicide attempt in adolescents. Colombia, 2016-2019

Characteristic	OR*	IC^†^ at 95%	P-value^‡^
Living in the municipal capital	1.423	1.385 to 1.462	0.000
Being a man	0.290	0.283 a 0.298	0.000

*OR = Odds Ratio; ^†^CI = Confidence Interval; ^‡^P-Value = Statistically Significant Value

When analyzing the prevalence and rate of attempted suicide in adolescents aged 12 to 17 years, an increasing trend is evident when observing the period from 2016 to 2019: in 2016 there was a rate of 11.7 cases *per* 10,000 inhabitants in constant increase until 2019, when this rate is 19.0 cases *per* 10,000 inhabitants (Table 3). When evaluating both the prevalence and the rate of attempted suicide in adolescents by sex from 2016 to 2019 the relationship present remains almost three times higher in female adolescents (Figure 1; Table 3).

**Table 3:** Prevalence of attempted suicide in adolescents aged 12 to 17 years in rate *per* 10,000 population. Colombia 2016-2019

DANE* population (12 and 17 years)				Prevalence in percentage			**Rate *per* 10,000 persons**		
Year	Male	Female	Total	Male	Female	Total	Male	Female	Total
2016	2,505,668	2,403,063	4,908,731	0.051	0.186	0.117	5.1	18.6	11.7
2017	2,486,050	2,383,822	4,869,872	0.077	0.265	0.169	7.7	26.5	16.9
2018	2,450,550	2,352,547	4,803,097	0.083	0.294	0.186	8.3	29.4	18.6
2019	2,455,036	2,357,586	4,812,622	0.091	0.293	0.190	9.1	29.3	19.0

*DANE = National Administrative Department of Statistics. For the calculation of these indicators, the 2005-2020 population projections by sex and age groups of the DANE were used as a reference population.

## Discussion

This research based on an ecological study provides the population diagnosis on the epidemiological behavior of suicide attempts from 2016 to 2019. Results evidence the health situation of the specific population group of Colombian adolescents, in different geographic areas and temporalities, around an event of interest in public health of high impact as is the suicide attempt. This diagnosis represents an important contribution for the planning of cross-sectoral actions that allow mitigating the phenomenon.

On the other hand, the study is relevant for the identification of needs in interdisciplinary care, and the mobilization of indispensable resources for the provision of health services, observation and follow-up of adolescents with suicidal ideation or attempts as a fundamental element to promote mental health and reduce the epidemiological data reported.

In the same way, it allows exposing research results in the area of Nursing, which enables greater visibility of the profession. Moreover, it shares the most relevant findings of the research, representing an input for the development of future projects and generation of effective interventions that impact on Public Health and Mental Health of the population.

According to WHO data for 2021, more than 700,000 people in the world die by suicide each year^9^, which is a topic of interest in Public Health due to its high prevalence, the economic burden it generates and the impact it has on the family and social environment^10^. Likewise, it represents the fourth leading cause of death among young people aged 15 to 19 years, which becomes really worrying because of the increasingly earlier age range, since for each suicide recorded, there are many more people who attempted suicide. Therefore, information on a previous suicide attempt becomes relevant, since it is the most important risk factor to foresee a future attempt^10^.

On the other hand, suicide is a complex and multifactorial phenomenon related to biological factors (such as gender)^11^, psychological (history of oppositional defiant disorder^12^, mental disorders, anxiety and depression)^13^, low socioeconomic and educational level (unemployment, limited access to basic necessities)^14^, cultural factors associated with abuse of alcohol^15^, psychoactive and psychotic substance (ethanol, *cannabis* ^16^, cocaine and amphetamines), and affective disorders, self-esteem problems^17^, physical and sexual abuse^15^, personal losses, destructive and violent events, physical illness and chronic pain^10^ ^,^ ^18^ ^-^ ^19^.

In relation to biological sex, this research reflects a behavior similar to that reported in another study developed in Barcelona, Spain^10^, which shows that women make more suicide attempts than men. In fact, from 2018 to 2019 twice as many episodes were documented by women, with no significant differences during the pandemic. However, there was an increase in consultations for suicidal ideation among the younger population possibly derived from confinement, which resulted in increased irritability, anxiety, depression and psychological distress due to increased exposure to the media and uncertainty about the future^20^ ^-^ ^21^.

Regarding the population’s conditions of vulnerability, according to the Profamilia Association and the United States Office of Foreign Disaster Assistance (OFDA-USAID)^22^, after the population of psychiatric centers, the population deprived of liberty and pregnant women, the Venezuelan migrant population is the fourth vulnerable population group with the highest number of suicide attempts in Colombia^22^. Regarding this last aspect, a publication with adolescents recruited in care centers for migrants in France highlights the difficulty to mentalize and verbalize emotions and feelings, as well as the difficulty to connect with others, feelings of loneliness and isolation that lead to negative thoughts about their lives^23^.

Associated with this situation, people in vulnerable situations or belonging to social minority populations show higher risk of suffering from mental disorders, since due to their minority status they are at a disadvantage in relation to the predominant or majority social sectors^24^. For example, migrants or people with scarce resources may present greater socioeconomic problems and greater deprivation of basic needs, which leads to thoughts of anguish and hopelessness that may be translated into self-harming actions.

On the other hand, it is important to pay attention to the group of pregnant women, since a study found that the prevalence of psychopathological antecedents, sadness, adjustment disorder with anxiety and depressed mood were risk factors that influenced a subsequent suicide attempt^25^.

In this regard, literature shows that it is prudent for different health professionals and the community in general to be alert to the possibility of suicidal ideation in adolescents who report feeling more irritable or worried than usual; who manifest negative feelings, such as pessimism, sadness, fear of the future, anxiety that additionally affect and interfere with daily life such as study, family life, concentration, sleep, food, hygiene and social contact.

Likewise, it is relevant to identify people who are consuming alcohol and/or psychoactive substances - as have additional problems^26^ -, who have thought of or have effectively self-harmed, or who are going through serious health problems, loss of a loved one, breakup of a couple; academic, family, economic or labor issues^19^, forced displacement, physical violence in its different dimensions, people who are part of child or adolescent protection centers or prisons or detention centers in general. These factors, individually or together, may favor self-injurious thoughts or actions.

For these reasons, the intervention of interdisciplinary groups of professionals who can recognize and act effectively in the management of mental pathologies is crucial. The mental pathologies include, but are not limited to: mood disorders, hallucinations, delusions, personality disorder, since according to the literature they have an impact on the prevention of suicidal behaviors, and can also help adolescents to take better alternatives to manage their problems^18^.

Along these lines, the work in educational institutions is one of the scenarios with greatest impact on the mental health of adolescents. Despite the fact that in some countries there are no services available for this population and that a significant proportion of adolescents do not attend school, professionals working in programs aimed at school health are often essential in identifying and addressing behavioral health problems, as well as in establishing the required steps to connect students and families with supporting systems for the promotion, prevention and management of situations related to suicide^27^.

In addition, a revaluation of health under a comprehensive and humanized approach is needed. This requires investment in training professionals who can assume the responsibility of promoting universal health coverage for the provision of friendly and affordable services to adolescents, especially in situations that violate their mental and physical health^28^.

On the other hand, it has been shown that Nursing professionals develop the ability to establish therapeutic relationships with people at risk of suicidal behavior and ideation, and to be active agents in identifying and assessing the needs of their users. Likewise, Nursing professionals have the ability to actively listen and obtain information from different sources to improve the understanding and health care required by the individual, as well as being active in mobilizing the resources required for the care, observation and follow-up of these individuals^18^.

Along the same lines, although the approach to adolescents at risk of suicide should be multidisciplinary, nursing professionals and especially mental health specialists play a crucial role in the detection of suicidal behavior and the promotion of healthy lifestyles. These professionals are competent to act with people at risk of suicide, helping them to reflect on the reasons that motivate them to make this decision. There is scientific evidence that some suicides could be prevented with a risk assessment and intervention and care by Nursing professionals^29^.

In this exercise of care by Nursing professionals, the Nursing Process (NP) is also presented as the working method of the discipline. It is an effective and evidence-based strategy for the development of actions consistent with the needs of people at risk or suicidal ideation.

Through the use of standardized languages one may identify and manage suicide-related situations. Among the diagnoses of the North American Nursing Diagnosis Association (NANDA)^30^ that can be applied to the topic studied are: Risk of suicidal behavior and Risk of suicidal conduct. On the other hand, care objectives can be planned in Nursing Outcomes Classification (NOC)^31^, such as: Suicide Risk Management and Self-Management of Suicidal Impulse.

In addition, Nursing Interventions Classifications (NIC)^32^ are indicated, such as suicide prevention and management of self-injurious behavior, representing a tangible way of approaching adolescents with these problems, and ensuring timely care according to their specific needs and based on referrals oriented to professionals involved in the fields of emergency and mental health care.

It is peremptory at this time when adolescents are experiencing an era characterized by a complex web of social relations that demands a committed and shared leadership between Nursing professionals and other health science professionals, oriented by the quality of interactions between health teams and the different components of the health system, including other sectors of social development in order to respond to the complexity of the health phenomena experienced by this population group in a rapid, effective and sustainable manner^33^, since there is a worldwide absence of specific mental health policies for children and adolescents, which may delay the care process and the prevention of suicidal behaviors^34^. Likewise, the discipline demands wise actions and decision-making focused on the Sustainable Development Goals^35^.

Among the limitations of this study, the limited information available regarding the quality and completeness of the reports is recognized, added with evaluation only of reported cases, lack of knowledge on other representative variables of the suicide attempt (family traits, school, background, etc.), and inability to control possible confounding bias are assumed as limitations, since logistic regression analysis could be performed reviewing multicollinearity, for not meeting the statistical requirements to develop it (global adjustment, goodness of fit and coefficients of determination). It is recommended to carry out intervention studies in the area of Nursing with a qualitative approach, in order to deepen the knowledge about the behavior of this phenomenon.

## Conclusion

Suicide attempts are constantly on the rise, being significantly manifested in the female sex and in adolescents from 15 to 17 years of age, corresponding to the subsidized regime, of black, mulatto and Afro-Colombian ethnicity, in addition to being residents of the municipal capital. In relation to vulnerability conditions, the most frequent are being in psychiatric centers, and in the care of the ICBF. Belonging to the male gender represents a protective factor that reduces suicide attempts in adolescents. It is worth considering, on the other hand, that in Colombia the suicide attempt as an event of interest to Public Health has been monitored since 2016, thus being very recent.

However, a gradual increase in the national incidence rate is observed since then. Finally, it has been evidenced that the Nursing professional has the ability to establish therapeutic relationships with adolescents at risk of suicidal ideation, in addition to representing an active agent in the identification and assessment of needs, with listening skills to obtain information to improve understanding and health care, enabling the implementation of psychological first aid and participation in psychotherapy activities.
